# Supplementary material for: The acute effect of wearable resistance load and placement upon change of direction performance in soccer players
Source: PLoS One. 2020 Nov 18;15(11):e0242493. doi: 10.1371/journal.pone.0242493 (PMC7673491; doi:10.1371/journal.pone.0242493)
Supplement: Attachment — Submitted filename: Comments to reviewers.docx [file pone.0242493.s002.docx]

Dear Editor,

We thank the reviewers for reviewing the manuscript. We have changed the manuscript according to the comments of the reviewers and hope that the changes we have made have improved the manuscript. We believe it is now suitable for your journal, and thus would appreciate it if this paper was considered for publication.

In the revised manuscript, we have used coloured text to indicate the major changes from the last version. Please see our responses and specifications of the changes/corrections made below.

This study was conducted without any funding from companies or manufacturers or outside organizations. Thereby the authors received no specific funding.

With kind regards,

Roland van den Tillaar Ph.D.

Please use the space provided to explain your answers to the questions above. You may also include additional comments for the author, including concerns about dual publication, research ethics, or publication ethics. (Please upload your review as an attachment if it exceeds 20,000 characters)

Reviewer #1: Well planned and conducted study into a growing area of research: WR. The COD aspect is a new area and adds to the sprint studies done to date.

Reviewer #2: Thank you for the opportunity to review this article.
This study aimed to examine the acute effect of different lower limb wearable resistance on placement (shank vs. thigh) and various loads (1−5% of body mass) upon change of direction (COD) ability.

Comments:
Introduction:
Page 9 line35-36: please add a reference.

We have added 3 references.

Line 40: football should be soccer

Changed now.

Page 12 line 110: please put a space between of and one.

Changed now.

Line 116 please change two minutes by 2 min, like the next sentence.

Changed it now.

Statistical analyses:
It should be better to convert partial eta squared to Cohens’d.

We have converted the partial eta squared to Cohen’s d

Results:

It is time to stop using the term “statistically significant” entirely. Nor should variants such as “significantly different,” “p < 0.05,” and “nonsignificant” survive, whether expressed in words, by asterisks in a table, or in some other way.’
<https://www.tandfonline.com/doi/pdf/10.1080/00031305.2019.1583913>

We have read the article and changed the significant difference in small, moderate and large effects as suggested by Hopkins et al. (2009). We still use asterisk in figure since it showed that these differences were larger than p < 0.05, but also had a large effect, which again is written in the text instead of significance. We have taken all significance wording away from the results and discussion parts.
